# Stunting, Poor Iron Status and Parasite Infection Are Significant Risk Factors for Lower Cognitive Performance in Cambodian School-Aged Children

**DOI:** 10.1371/journal.pone.0112605

**Published:** 2014-11-18

**Authors:** Marlene Perignon, Marion Fiorentino, Khov Kuong, Kurt Burja, Megan Parker, Sek Sisokhom, Chhoun Chamnan, Jacques Berger, Frank T. Wieringa

**Affiliations:** 1 Institut de Recherche pour le Développement, Montpellier, France; 2 Department of Fisheries Post-Harvest Technologies and Quality Control, Ministry of Agriculture, Forestry and Fisheries, Phnom Penh, Cambodia; 3 United Nations World Food Programme, Phnom Penh, Cambodia; 4 PATH, Seattle, Washington, United States of America; 5 Department of Psychology, Royal University of Phnom Penh, Phnom Penh, Cambodia; CSIR-Institute of Genomics and Integrative Biology, India

## Abstract

**Background:**

Nutrition is one of many factors affecting the cognitive development of children. In Cambodia, 55% of children <5 y were anemic and 40% stunted in 2010. Currently, no data exists on the nutritional status of Cambodian school-aged children, or on how malnutrition potentially affects their cognitive development.

**Objective:**

To assess the anthropometric and micronutrient status (iron, vitamin A, zinc, iodine) of Cambodian schoolchildren and their associations with cognitive performance.

**Methods:**

School children aged 6–16 y (n = 2443) from 20 primary schools in Cambodia were recruited. Anthropometry, hemoglobin, serum ferritin, transferrin receptors, retinol-binding protein and zinc concentrations, inflammation status, urinary iodine concentration and parasite infection were measured. Socio-economic data were collected in a sub-group of children (n = 616). Cognitive performance was assessed using Raven’s Colored Progressive Matrices (RCPM) and block design and picture completion, two standardized tests from the Wechsler Intelligence Scale for Children (WISC-III).

**Results:**

The prevalence of anemia, iron, zinc, iodine and vitamin A deficiency were 15.7%; 51.2%, 92.8%, 17.3% and 0.7% respectively. The prevalence of stunting was 40.0%, including 10.9% of severe stunting. Stunted children scored significantly lower than non-stunted children on all tests. In RCPM test, boys with iron-deficiency anemia had lower scores than boys with normal iron status (−1.46, p<0.05). In picture completion test, children with normal iron status tended to score higher than iron-deficient children with anemia (−0.81; p = 0.067) or without anemia (−0.49; p = 0.064). Parasite infection was associated with an increase in risk of scoring below the median value in block design test (OR = 1.62; p<0.05), and with lower scores in other tests, for girls only (both p<0.05).

**Conclusion:**

Poor cognitive performance of Cambodian school-children was multifactorial and significantly associated with long-term (stunting) and current nutritional status indicators (iron status), as well as parasite infection. A life-cycle approach with programs to improve nutrition in early life and at school-age could contribute to optimal cognitive performance.

## Introduction

Worldwide, undernutrition and micronutrient deficiencies substantially impair human health and socio-economic development. Both developed and developing countries are concerned by the burden of micronutrient deficiencies disorders, although the highest prevalence exist in Sub-Saharan Africa and South Asia [1]. In Cambodia, despite a considerable reduction in national poverty since the mid-1990s, undernutrition remains a major problem, and approximately one fifth of the population was still living on less than $1.25 per day in 2009. According to the most recent Cambodia Demographic and Health Survey (CDHS 2010), 40% of children are stunted, 11% are wasted, and 55% of children of 6–59 months are anemic [2].

Nutrition is one of many key factors affecting mental development of children. Deficiencies of critical micronutrients such as iron, folate or iodine can lead to impaired cognitive functions due to their decisive role in brain development. For example, iron deficiency (ID) has been estimated to impair the optimal mental development of 40% to 60% of the developing world’s infants [3]. Indirectly, deficiencies of other micronutrients like vitamin A or zinc can increase the risk of morbidity thereby increasing school absenteeism and reducing learning abilities and school performance.

Iron deficiency anemia (IDA), the most severe degree of ID, has been associated with poorer cognitive performance but it is still unclear if malnutrition has detrimental effects on cognitive performance independent of anemia in school-aged children [4]. Some recent systematic reviews based on randomized controlled trials provided evidence for a positive effect of iron supplementation on different measures of cognition in anemic and non-anemic children >5 y [5], on attention and concentration in adolescents and women [6], and beneficial effect of micronutrient interventions (food-based or supplementation) on short term memory [7]. In addition to IDA, stunting is a known risk factor for impaired child development [8]. Despite a general decrease of the global prevalence, stunting still affects one third of the children under 5 y in the developing world [9]. Several studies have documented the relationship between cognitive ability and stunting in young children [10], [11] but few have explored it within school-aged children [12]–[14].

School-aged children are often omitted from public health research. Early child development programs typically focus on the nutritional status and milestones of children under 5 y. Thus, our knowledge regarding the prevalence and impact of micronutrient deficiencies on children >5 y remains scarce. Yet, some areas of the brain and higher cognitive functions continue to develop throughout childhood and adolescence [15]. The myelination of frontal lobes which are thought to be responsible for executive, “higher-order” cognitive activities, starts around 6 mo and continues until adulthood. Changes in the volumes of cortical gray and white matters, significantly correlated to children’s performance on a verbal learning task, occur during childhood and adolescence [16]. As during infancy, this ongoing brain development throughout childhood is likely to be affected by detrimental effect of poor nutritional status, hence warranting further research on the impact of nutritional status on cognitive performance of school-aged children. In Cambodia, there is currently no data available concerning the nutritional status of children >5 y, nor how malnutrition potentially affects their mental development.

The present study aimed to evaluate the anthropometric and micronutrient status (iron, vitamin A, zinc and iodine) of Cambodian children aged 6–16 y and to determine if these outcomes are associated with cognitive performance.

## Subjects and Methods

### Study population and design

Data were collected as part of a randomized placebo-controlled trial investigating the impact of multi-micronutrient fortified rices on health and development of Cambodian schoolchildren (FORISCA UltraRice+NutriRice study, FOrtified RIce for School meals in CAmbodia). Baseline data collection was conducted in November 2012 in 20 primary schools from 5 districts of Kampong Speu province in Cambodia. Kampong Speu is one of the 23 provinces of Cambodia, situated 60 km west of the capital Phnom Penh. Agriculture is predominant, with rice farming as the main occupation and income source. The schools were randomly selected from primary schools participating in school meal or take-home ration programs of the UN World Food Program (WFP). Children attending the selected schools were eligible to be part of the study if they were between 6–16 y of age, had written informed consent from parents/caregivers and did not have any mental or severe physical handicap. In each school, 132 children were randomly selected after stratification by sex and grade, hence 2640 children. 197 children were not recruited because they were absent on the day of data collection or refused to participate. Hence, a total of 2443 schoolchildren aged 6–16 y participated in the study. The study was approved by the National Ethic Committee for Health Research (NECHR) of the Ministry of Health, Phnom Penh, Cambodia, the Ministry of Education, Youth and Sports, Phnom Penh, Cambodia, and the Research Ethics Committee of PATH, Seattle, USA.

### Anthropometric measurements

Height and weight were measured using standardized procedures [17], [18]. Children wore minimum clothing and no shoes. Height was measured in duplicate to the nearest 0.1 cm with a wooden stadiometer. Weight was measured once, to the nearest 0.1 kilogram using an electronic scale (Seca, 881 U, Germany). Z-scores for height-for-age (HAZ) were calculated with AnthroPlus software version 1.0.4 using the WHO 2007 standards. Moderate and severe stunting were respectively defined as −3<HAZ<−2 and HAZ<−3 respectively.

### Cognitive performance tests

The cognitive performance evaluation included three tests: the Raven’s Colored Progressive Matrices test (RCPM), and two standardized tests from the Wechsler Intelligence Scale for Children (WISC III): block design and picture completion. RCPM, the coloured form of Raven’s Progressive Matrices test for use with children, is a widely used nonverbal test of intelligence which was designed as a measure of overall intellectual ability [19], [20]. The WISC III, designed for children aged 6–16 y, is one of the most widely used tests of the intelligence of children [21]. Block design is a measure of problem solving to assess executive functions [21]. Picture completion evaluates alertness to detail and visual discrimination. The cognitive tests were conducted by 25 students from Psychology department of Royal University of Phnom Penh. The interviewers were trained in a 1 week workshop to ensure standardization in the assessment and scoring procedures. Each child was tested individually using standardized test protocols translated into Khmer language. Since norms are not available for Cambodia, interpretations of the scores of cognitive test were used as raw scores. For all cognitive tests, higher scores indicate better performance.

### Blood and urine samples collection

Blood samples (5 ml) were collected by venipuncture and aliquoted in a trace-element free vacutainers with no anticoagulant (Vacuette, Greiner Bio One, Austria). Urine samples were collected from the children in a sterile plastic container. Blood and urine samples were then stored in cool-boxes containing ice-packs and transported to Phnom Penh within 5 h of collection. The blood samples were centrifuged at 2700 rpm (1300 g) for 10 min at room temperature. Serum and urine were then aliquoted in capped Eppendorf tubes and stored at −30°C until transfer for analysis.

### Hemoglobin concentration

Hemoglobin concentrations were determined immediately after blood taking using the HemoCue (301+ system, HemoCue Angholm, Sweden). Anemia was defined as hemoglobin concentration <115 g/L for children between the ages 6 and 11 y, <120 g/L for children between the ages 12 and 14 y and girls aged 15 y and older and <130 g/L for boys aged 15 y and older according to WHO guidelines [22].

### Laboratory analysis

#### Ferritin (FER), soluble transferrin Receptor (TfR), retinol-binding protein (RBP), C-reactive protein (CRP), α1-acid-glycoprotein (AGP) and zinc serum concentrations

Serum samples were sent on dry ice to the VitMin laboratory (Willstaett, Germany) for determination of retinol-binding protein (RBP), C-reactive protein (CRP), ferritin (FER), soluble transferrin receptor (TfR) and α1-acid-glycoprotein (AGP), and to National Institute of Nutrition (Hanoi, Vietnam) for zinc analysis. RBP, FER, TfR, CRP, AGP were measured by a sandwich enzyme-linked immunosorbent assay (ELISA) technique [23]. Zinc concentration was measured using a flame atomic absorption spectrophotometer (GBC, Avanta+) using trace element-free procedures. Inflammation was defined as high CRP (>5 mg/L) and/or high AGP concentrations (>1 g/L). Inflammation status was then categorized in four groups based on CRP and AGP levels: no inflammation (normal CRP and AGP), incubation (high CRP and normal AGP), early convalescence (high CRP and AGP), and late convalescence (normal CRP and high AGP) [24]. FER is affected by presence of infection or inflammation, therefore FER concentrations were adjusted using correction factors published by Thurnham et al., namely 0.77, 0.53 and 0.75 for children respectively in incubation, early convalescence, and late convalescence phases. Low FER (corrected value <15 µg/L) was used as indicator of depleted iron stores [22], and high TfR (>8.3 mg/L) as indicator of tissue iron deficiency [25], [26]. Iron deficiency was defined using both FER and TfR indicators, i.e. by depleted iron stores or tissue iron deficiency. Total body iron (BI) was calculated from FER corrected for inflammation and TfR as described by Cook *et al.* [27]. Serum retinol is bound to RBP in a 1-to-1 complex, hence RBP concentrations were used to evaluate vitamin A status [28]. RBP concentrations were adjusted for the presence of inflammation using correction factors of 1.15, 1.32 and 1.12 for incubation, early convalescence and late convalescence phases respectively [29]. Corrected RBP cut-offs were used to define marginal vitamin A status (0.70 µmol/L ≤ corrected RBP<1.05 µmol/L), vitamin A deficiency (<0.70 µmol/L) and severe vitamin A deficiency (<0.35 µmol/L) respectively [28], [30]. Zinc deficiency was defined using the following cut-offs: serum zinc concentration <0.66 mg/L for girls ≥10 y, <0.70 mg/L for boys ≥10 y, and <0.65 mg/L for boys and girls <10 y [31].

#### Urinary iodine concentration

Urine samples were sent on dry ice to the Provincial Preventive Medicine Center (Thai Nguyen, Vietnam) for determination of urinary iodine concentration (UIC) using spectrophotometric methods [32]. Iodine deficiency (IDD) was defined by a median UIC below 100 µg/L and/or a proportion of participants below 50 µg/L higher than 20%, and iodine nutrition above requirements by a median UIC above 200 µg/L [33].

### Parasite infestation

Plastic containers and instructions for fecal sample collection were distributed to the children on the day of data collection and requested to be returned with fecal sample to the school the following day. Samples were then stored in a cool box, transported to the National Malaria Center (CNM, Phnom Penh, Cambodia) and stored at 4°C until analysis. Quantitative parasite egg counts were performed by CNM using the Kato-Katz method [34]. The egg output was expressed as eggs per gram feces (epg).

### Socio-economic survey

Socio-economic information was collected on a sub-sample (n = 616 children, FORISCA-NutriRice study) by trained interviewers during household visits. Questionnaires were answered by parents or caretaker and included information about household characteristics, caretaker’s level of education and amount and source of household income.

### Data management and statistical analysis

Data entry, including quality checks and validation by double entry of questionnaires, was performed with EpiData version 3.1 (EpiData Association, Odense, Denmark). Data management and analyses were performed using SPSS version 20.0 software (SPSS, Inc., Chicago, IL). Normality of distributions was evaluated using Kolmogorov-Smirnoff test. Not normally distributed data were considered to be reasonably close to normality to allow parametric tests when skewness and kurtosis values ranged between −1.0 and +1.0. Continuous variables that were not normally distributed were log-transformed. Block design score was categorized as score below and above median value. All analyses took into account characteristics of the cluster sampling design (school clusters). The main effects of explanatory variables on cognitive tests scores were first assessed in univariate analysis using ANCOVA for RCPM and Picture completion tests, and logistic regression for Block design test, with all analysis including age as covariate. Multivariate analyses were then performed to evaluate the associations between cognitive tests scores and variables assessing nutritional status (stunting, anemia and micronutrient status) while taking into account the effect modification or confounding of other variables (age, gender, parasite infection, socio-economic status). Any variable having a p-value <0.25 in the univariate test was considered for the multivariate analysis. Interactions were tested and if significant at the 0.05 level, analyses were run separately at each level of the variable modifying the effect. Multiple comparisons were conducted by using the Bonferroni post-hoc test.

## Results

### Characteristics of the studied sample

A total of 2443 children from grades 1–6 participated in the study. Characteristics of the participants are presented in **Table 1**. The mean ± SD age of children was 9.6±2.3 y and half (49.9%) were girls. The prevalence of anemia was 15.7% including 0.1% severe anemia. There was no significant difference in anemia prevalence between boys and girls, or age groups; however, prevalence varied between 4.8% and 30.0% across the schools. The overall prevalence of stunting (HAZ<−2SD) was 40.0%, including 10.9% severe stunting (HAZ<−3SD). Children ≥10 y were significantly more affected by stunting (54.0% and 29.6% respectively) or severe stunting (18.5% and 5.3% respectively) than children <10 y, but no difference was found between gender. Inflammation (CRP>5 mg/L and/or AGP>1 g/L) was found in more than one third of the children (39.5%), with boys being more affected than girls. Parasite infection was found in 18% of the children, boys being more affected than girls (p<0.05). Only 1.5% of the children had depleted iron stores (FER<15 µg/L) whereas 50.1% had tissue iron deficiency (TfR>8.3 mg/L). There was no significant difference between age groups or genders for both indicators. Prevalence of iron deficiency, as defined by low FER and/or high TfR, was 51.2% without any difference between age groups or gender. Only 2% of the children had negative body iron stores but marginal body iron stores (total body iron <4 mg/kg body weight) were prevalent (13.9%). Prevalence of iron-deficiency anemia was 10.0% with no difference between boys and girls. Prevalence of vitamin A deficiency (VAD) was 0.7%, with no severe VAD. However, 7.9% of the children had marginal VA status (0.7≤corrected RBP<1.05 µmol/L). Children <10 y were significantly more affected by marginal VA status (9.4% and 6% respectively) than children >10 y (p<0.05). Prevalence of marginal VA status was also higher in boys than in girls (9.2% and 6.6% respectively, p<0.05). Most children (93%) exhibited low serum zinc concentrations, indicative of zinc deficiency. Approximately one fifth (17%) of the children were iodine deficient while half (50.2%) had iodine intake above requirements.

**Table 1:** Characteristics of school-children participating in the study.

	BOYS	GIRLS	ALL	p-value
n	1223	1220	2443	
Age	9.75±2.34	9.54±2.17	9.65±2.26	p<0.05
% inflammation	41.6 (n = 491)	37.5 (n = 446)	39.5 (n = 937)	p<0.05
% parasite infection	20.0 (n = 177)	16.1 (n = 148)	18.0 (n = 325)	p<0.05
**ANTHROPOMETRY**				
HAZ	−1.80±1.00 (n = 1219)	−1.71±1.06 (n = 1216)	−1.75±1.03 (n = 2435)	p<0.05
% HAZ<−2SD	41.6 (n = 507)	38.5 (n = 468)	40.0 (n = 965)	NS
% HAZ<−3SD	11.1 (n = 135)	10.8 (n = 131)	10.9 (n = 266)	NS
**IRON STATUS**				
Hb (g/L)	123.8±9.9 (n = 1203)	124.5±9.5 (n = 1206)	124.2±9.7 (n = 2409)	NS
% anemia	16.8 (n = 202)	14.7 (n = 177)	15.7 (n = 379)	NS
% severe anemia	0.1 (n = 1)	0.2 (n = 2)	0.1 (n = 3)	
FER^1,2^ (µg/L)	66.28±1.68 (n = 1181)	68.63±1.68 (n = 1190)	67.45±1.68 (n = 2371)	NS
% FER^1^ <15 µg/L	1.9 (n = 22)	1.2 (n = 14)	1.5 (n = 36)	NS
TfR^2^ (mg/L)	8.56±1.32 (n = 1181)	8.41±1.30 (n = 1190)	8.48±1.31 (n = 2371)	NS
% TfR >8.3 mg/L	51.8 (n = 612)	50.3 (n = 598)	51.0 (n = 1210)	NS
% ID^3^ total	52.2 (n = 616)	50.3 (n = 599)	51.2 (n = 1215)	NS
% ID^3^ with anemia	10.8 (n = 127)	9.3 (n = 111)	10.0 (n = 238)	NS
Body iron (mg/kg)	5.90±2.27 (n = 1181)	6.09±2.21 (n = 1190)	5.99±2.24 (n = 2371)	p<0.05
% body iron <0	2.5 (n = 29)	1.6 (n = 19)	2.0 (n = 48)	NS
% body iron <2	5.0 (n = 59)	5.0 (n = 59)	5.0 (n = 118)	NS
% body iron <4	14.5 (n = 171)	13.3 (n = 158)	13.9 (n = 329)	NS
**VITAMIN A STATUS**				
RBP^1^ (µmol/L)	1.54±0.41 (n = 1181)	1.62±0.45 (n = 1190)	1.58±0.43 (n = 2371)	p<0.01
% marginal VA status^4^	9.2 (n = 109)	6.6 (n = 79)	7.9 (n = 188)	p<0.05
% VAD^5^	0.8 (n = 10)	0.6 (n = 7)	0.7 (n = 17)	NS
**IODINE STATUS**				
% iodine deficiency^6^	14.8 (n = 175)	19.8 (n = 233)	17.3 (n = 408)	p<0.05
% above requirements^7^	52.5 (n = 620)	47.9 (n = 564)	50.2 (n = 1184)	p<0.05
**ZINC STATUS**				
% zinc deficiency^8^	93.7 (n = 931)	91.9 (n = 907)	92.8 (n = 1838)	NS
**SOCIO-ECONOMIC STATUS (on a sub-group n = 616)**				
Income^9^ ($/year)	1995 (495; 4291)	1825 (500; 4050)	1935 (500; 4150)	
Caretaker's level of education:				
% no or informal schooling	11.9 (n = 35)	14.8 (n = 42)	13.3 (n = 77)	NS
% primary school	65.3 (n = 192)	59.0 (n = 167)	62.2 (n = 359)	NS
% secondary school	22.8 (n = 67)	26.1 (n = 74)	24.4 (n = 141)	NS

Results are mean ± SD unless stated, ^1^corrected for inflammation,^ 2^geometric mean ± SD, ^3^based on FER^1^ <15 µg/L and/or TfR >8.3 mg/L, ^4^ 0.7≤ RBP^1^ <1.05 µmol/L, ^5^RBP^1^ <0.7 µmol/L, ^6^UIC <100 µg/L, ^7^UIC ≥200 µg/L, ^8^zinc <0.66 mg/L for girls ≥10 y, <0.70 mg/L for boys ≥10 y, and <0.65 mg/L for boys and girls <10 y, ^9^median (10th; 90th), HAZ: height-for-age z-scores, Hb: hemoglobin, FER: ferritin, TfR: transferrin receptors, ID: iron-deficiency, NS: not significant, RBP: retinol binding protein, VA: vitamin A, VAD: vitamin A deficiency.

### Factors associated with cognitive performance

Cognitive scores were associated with stunting and micronutrient status (**Table 2** (RCPM test), **Table 3** (Picture completion test) and **Table** **4** (Block design test)). Children with severe stunting scored significantly lower than non stunted children in all tests (p<0.001 for all), with reduction of score reaching up to −2 points in RCPM test (12% of mean score), or −1.7 points in picture completion test (22% of mean score) after adjustment on all variables. Children with moderate stunting scored significantly lower (Picture completion and Block design tests, both p<0.001) or tend to score lower (RCPM test, p = 0.053) when compared with non-stunted children. For the block design test, severely stunted children were 2.5 times more likely to score below the median value than children with normal height status (OR = 2.53, p<0.001).

**Table 2:** Factors associated with cognitive performance in RCPM test among participating school children.

Variable	n	Estimatedmean^1^± SE	Estimated meandifference^1^ (95% CI)	p-value	Estimated meandifference (95% CI)adjusted for allvariables^9^		p-value	
**Gender**								
male	1201	16.88±0.14						
female	1224	16.44±0.14	−0.44 (−0.77; −0.12)	0.008	−0.72 (−1.12; −0.33)		p<0.001	
**Stunting**								
normal status	1500	17.03±0.13						
−3< HAZ <−2	677	16.41±0.26	−0.62 (−1.32; 0.08)	0.10	−0.56 (−1.12; 0.006)		0.053	
HAZ <−3	244	15.12±0.29	−1.91 (−2.70; −1.12)	p<0.001	−1.97 (−2.81; −1.12)		p<0.001	
**Parasite infection**								
not infected	1471	17.49±0.11						
infected	324	17.00±0.28	−0.49 (−1.09; 0.11)	0.11	M	0.29 (−0.49; 1.08)		0.47
					F	−1.43 (−2.18; −0.68)		0.016
**Iron deficiency^2^**								
no ID	1148	17.21±0.13						
ID without anemia	968	16.93±0.14	−0.28 (−0.73; 0.18)	0.43	M	−0.31 (−1.10; 0.48)		1.00
					F	−0.31 (−1.01; 0.38)		0.84
ID with anemia	236	16.41±0.35	−0.81 (−1.70; 0.086)	0.092	M	−1.46 (−2.73; −0.18)		0.019
					F	−0.36 (−1.56; 0.84)		1.00
**FER^3^ (mg/L)**								
≥50	1811	17.19±0.10						
15–50	507	16.69±0.20	−0.50 (−1.03; 0.02)	0.068				
<15	34	16.50±1.07	−0.69 (−3.26; 1.87)	1.00				
**TfR (mg/L)**								
≤8.3	1152	17.21±0.13						
>8.3	1200	16.86±0.12	−0.348 (−0.698; 0.01)	0.052				
**Body iron (mg/kg)**								
>4	2028	17.16±0.09						
0–4	278	16.42±0.28	−0.75 (−1.44; −0.06)	0.029				
<0	46	15.85±0.82	−1.32 (−3.29; 0.65)	0.33				
**VA status**								
normal VA status	2170	16.69±0.12						
marginal VAstatus^4^	173	16.48±0.42	−0.21 (−1.06; 0.65)	0.63				
VAD^5^	15	16.15±1.26	−0.54 (−3.02; 1.95)	0.67				
**Iodine status**								
adequate	763	17.21±0.18						
iodine deficiency^6^	406	16.72±0.33	−0.49 (−1.39; 0.41)	0.57				
above requirements^7^	1171	17.33±0.14	1.12 (−0.42; 0.65)	1.00				
**Zinc status**								
normal status	140	17.62±0.51						
zinc deficiency^8^	1827	17.08±0.11	−0.54 (−1.57; 0.49)	0.30				

1 adjusted for age, ^2^defined by FER^3^<15 µg/L and/or TFR >8.3 mg/L, ^3^corrected for inflammation, ^4^0.7≤ RBP^3^ <1.05 µmol/L, ^5^RBP^3^ <0.7 µmol/L, ^6^UIC <100 mg/L, ^7^UIC ≥200 mg/L, ^8^zinc <0.66 mg/L for girls ≥10 y, <0.70 mg/L for boys ≥10 y, and <0.65 mg/L for boys and girls <10 y, ^9^significant interactions between gender and parasite, and gender and iron deficiency, ID: iron deficiency, FER: ferritin, TfR: transferrin receptors, VA: vitamin A, VAD: VA deficiency, M: male, F: female, RCPM: Raven’s colored progressive matrices.

**Table 3:** Factors associated with cognitive performance in Picture completion test among participating school children.

Variable	n	Estimatedmean^1^± SE	Estimated meandifference^1^ (95% CI)	p-value	Estimated meandifference (95% CI)adjusted for allvariables^9^		p-value
**Gender**							
male	1201	7.38±0.13					
female	1224	7.29±0.14	−0.09 (−0.41; 0.23)	0.60			
**Stunting**							
normal status	1500	7.80±0.13					
−3< HAZ <−2	677	6.69±0.25	−1.11 (−1.79; −0.43)	p<0.001	−1.07 (−1.62; −0.52)		p<0.001
HAZ <−3	244	5.99±0.29	−1.81 (−2.58; −1.04)	p<0.001	−1.68 (−2.50; −0.86)		p<0.001
**Parasite infection**							
not infected	1485	7.69±0.18					
infected	304	7.09±0.31	−0.60 (−1.30; 0.10)	0.095	M	0.02 (−0.73; 0.77)	0.96
					F	−0.85 (−1.61; −0.09)	0.028
**Iron deficiency^2^**							
no ID	1148	7.43±0.17					
ID without anemia	968	7.24±0.18	−0.19 (−0.78; 0.40)	1.00	−0.49 (−1.01; 0.02)		0.064
ID with anemia	236	7.00±0.36	−0.43 (−1.39; 0.52)	0.82	−0.81 (−1.66; 0.04)		0.067
**FER^3^ (mg/L)**							
≥50	1811	7.89±0.10					
15–50	507	7.53±0.19	−0.36 (−0.88; 0.15)	0.27			
<15	34	8.11±1.05	0.22 (−2.31; 2.74)	1.00			
**TfR (mg/L)**							
≤8.3	1152	7.98±0.12					
>8.3	1200	7.64±0.12	−0.35 (−0.69; −0.01)	0.048			
**Body iron (mg/kg)**							
>4	2028	7.90±0.09					
0–4	278	7.13±0.27	−0.77 (−1.45; −0.10)	0.019			
<0	46	7.33±0.80	−0.57 (−2.51; 1.36)	1.00			
**VA status**							
normal VA status	2170	7.36±0.11					
marginal VA status^4^	173	7.56±0.41	0.19 (−0.82; 1.21)	1.00	0.08 (−0.84; 0.99)		1.00
VAD^5^	15	4.79±1.22	−2.57 (−5.52; 0.37)	0.11	−0.86 (−3.43; 1.71)		1.00
**Iodine status**							
adequate	763	7.73±0.17					
iodine deficiency^6^	406	7.72±0.33	−0.01 (−0.90; 0.88)	1.00			
above requirements^7^	1171	8.03±0.14	0.30 (−0.23; 0.83)	0.51			
**Zinc status**							
normal status	140	8.21±0.50					
zinc deficiency^8^	1827	7.83±0.10	−0.38 (−1.38; 0.62)	0.45			

1 adjusted for age, ^2^defined by FER^3^ <15 µg/L and/or TFR >8.3 mg/L, ^3^corrected for inflammation, ^4^0.7≤ RBP^3^ <1.05 µmol/L, ^5^RBP^3^ <0.7 µmol/L, ^6^UIC <100 mg/L, ^7^UIC ≥200 mg/L, ^8^zinc <0.66 mg/L for girls ≥10 y, <0.70 mg/L for boys ≥10 y, and <0.65 mg/L for boys and girls <10 y, ^9^significant interactions between gender and parasite, ID: iron deficiency, FER: ferritin, TfR: transferrin receptors, VA: vitamin A, VAD: vitamin A deficiency, M: male, F: female.

**Table 4:** Univariate and multivariate analysis of factors associated with poor performance in Block design test among participating schoolchildren.

Variable	n	odds-ratio^1^(95% CI)	p-value	odds-ratio adjusted forall variables (95% CI)	p-value
**Gender**					
male	1208	1		1	
female	1213	1.28 (1.06; 1.55)	0.009	1.27 (0.99; 1.63)	0.058
**Stunting**					
normal status	1446	1		1	
−3< HAZ <−2	706	1.88 (1.50; 2.37)	p<0.001	1.73 (1.29; 2.33)	p<0.001
HAZ <−3	263	3.67 (2.56; 5.26)	p<0.001	2.35 (1.47; 3.75)	p<0.001
**Parasite infection**					
not infected	1471	1		1	
infected	324	1.64 (1.21; 2.24)	0.002	1.73 (1.24; 2.42)	0.001
**Iron deficiency^2^**					
no ID	1148	1		1	
ID without anemia	968	0.95 (0.77; 1.17)	0.62	1.09 (0.83; 1.43)	0.52
ID with anemia	236	1.32 (0.95; 1.85)	0.10	1.17 (0.74; 1.85)	0.26
**FER^3^ (mg/L)**					
≥50	1811	1			
15–50	507	1.11 (0.88; 1.40)	0.39		
<15	34	1.31 (0.60; 2.90)	0.50		
**TfR (mg/L)**					
≤8.3	1152	1			
>8.3	1200	1.01 (0.83; 1.23)	0.93		
**Body iron (mg/kg)**					
>4	2028	1			
0–4	278	1.23 (0.92; 1.66)	0.17		
<0	46	1.24 (0.62; 2.49)	0.54		
**VA status**					
normal VA status	2149	1			
marginal VA status^4^	186	0.91 (0.64; 1.30)	0.60		
VAD^5^	17	1.67 (0.48; 5.89)	0.42		
**Iodine status**					
adequate	763	1			
iodine deficiency^6^	406	0.91 (0.68; 1.21)	0.52		
above requirements^7^	1171	0.94 (0.74; 1.19)	0.60		
**Zinc status**					
normal status	140	1		1	
zinc deficiency^8^	1827	1.43 (0.95; 2.15)	0.091	1.32 (0.82; 2.13)	0.26

1 adjusted for age, ^2^defined by FER^3^ <15 µg/L and/or TFR >8.3 mg/L, ^3^corrected for inflammation, ^4^0.7≤ RBP^3^ <1.05 µmol/L, ^5^RBP^3^ <0.7 µmol/L, ^6^UIC <100 mg/L, ^7^UIC ≥200 mg/L, ^8^zinc <0.66 mg/L for girls ≥10 y, <0.70 mg/L for boys ≥10 y, and <0.65 mg/L for boys and girls <10 y, ID: iron deficiency, FER: ferritin, TfR: transferrin receptors, VA: vitamin A, VAD: vitamin A deficiency, M: male, F: female.

Univariate analysis showed that positive but marginal body iron stores (0<body iron ≤4 mg/kg) were associated with significantly lower scores in RCPM and picture completion tests (both p<0.05). After adjustment on all variables, boys with iron-deficiency anemia (IDA) scored significantly lower than children with normal iron status in RCPM test (−1.46; p<0.05). Score difference was not significant for girls with IDA. For the picture completion test, children with normal iron status tended to score higher than iron-deficient children with anemia (−0.81; p = 0.067) or without anemia (−0.49; p = 0.064), for both genders. Vitamin A deficiency, iodine disorders (deficiency or excessive intake) and zinc deficiency were not significantly associated with cognitive performance.

Girls scored significantly lower than boys in the RCPM test (−0.72; p<0.01) and were more likely (OR = 1.27; p<0.05) to score below the median value in the block design test. Parasite infection was associated with an increased risk of scoring below median value for the block design test (OR = 1.62; p<0.05), and with lower scores in RCPM and picture completion tests, but only for girls (both p<0.05).

In the sub-sample of school children for which data were available (n = 616), socio-economic status (caretaker’s level of education and household’s income) was significantly associated with scores in RCPM test, but not in Picture completion and Block design tests. In RCPM test, higher household’s income and caretaker’s level of education were both associated with higher test score (both p<0.05, **Table 5**). Results of multivariate analysis of association between RCPM scores and explanatory variables before and after adjustment on socio-economic status are given in **Table 5**. The results obtained for this sub-group were similar to those obtained on all children, with lower statistical significance as a consequence of smaller number of subjects. Adjustment on socio-economic status did not change the results: severe stunting and parasite infection were associated with lower scores in RCPM test, as well as IDA for boys.

**Table 5:** Factors associated with cognitive performance in RCPM test before and after adjustment on socio-economic status in a sub-sample of school children (n = 616).

Variable	n	mean difference^1^(95% CI)		p-value	mean difference^2, 3^ (95% CI)after adjustment for SES		p-value
**Gender**							
male	311						
female	305	−0.29 (−1.06; 0.48)		0.47	−0.47 (−1.27; 0.33)		0.25
**Stunting**							
normal status	367						
−3< HAZ <−2	176	−0.33 (−1.42; 0.75)		1.00	−0.55 (−1.67; 0.57)		0.71
HAZ <−3	67	−1.68 (−3.33; −0.04)		0.044	−1.60 (−3.29; 0.08)		0.067
**Parasite** **infection**							
not infected	387						
infected	48	M	−0.09 (−1.85; 1.66)	0.92	M	0.20 (−1.63; 2.03)	0.83
		F	−2.32 (−4.24; −0.40)	0.018	F	−2.27 (−4.25; −0.29)	0.025
**Iron** **deficiency^4^**							
no ID	261						
ID withoutanemia	262	M	0.13 (−1.49; 1.74)	1.00	M	−0.01 (−1.63; 1.65)	1.00
		F	−0.25 (−1.75; 1.24)	1.00	F	−0.26 (−1.77; 1.25)	1.00
ID with anemia	65	M	−2.09 (−4.60; 0.42)	0.14	M	−2.23 (−4.78; 0.32)	0.11
		F	−1.05 (−3.25; 1.15)	0.76	F	−0.95 (−3.31; 1.41)	1.00
**Socio-economic** **status**							
Householdincome^5^	616					0.03 (0.00; 0.05)	0.048
Caretaker’seducation^6^	616					0.18 (0.02; 0.34)	0.024

1 variables in the model: age, gender, stunting, parasite infection, iron deficiency, ^2^variables in the model: age, gender, stunting, parasite infection, iron deficiency, caretaker's level of education, income, ^3^interaction between gender and parasite infection, and gender and iron-deficiency,^ 4^defined by FER (corrected for inflammation) <15 µg/L and/or TFR >8.3 mg/L, ^5^x100 US$/year, ^6^years of schooling, ID: iron deficiency, SES: socio-economic status, M: male, F: female.

## Discussion

Within this large cross-sectional survey of Cambodian school children, both long-term nutritional deficits as reflected by stunting and current micronutrient status, especially iron status, significantly affected cognitive performance. According to the WHO classification system, the observed prevalence of anemia (15.7%) indicated a mild public health problem in Cambodia, with only 3 cases of severe anemia. The prevalence of depleted iron stores (1.5%) or negative body iron (2%) were both very low. In contrast, more than half of the children had high TFR concentrations. TFR reflects tissue iron needs but may have also been increased by factors like hemoglobinopathy or inflammation [35], [36]. TFR levels were actually higher in children showing abnormal Hb type (HbE) or inflammation, but prevalence of high TFR was still important in the sub-sample of children with normal Hb profile (48.5%) or without inflammation (42.1%). Iron stores being adequate for most of the children (as indicated by the high ferritin concentrations), high TfR concentration suggests a functional tissue iron deficiency due to either an impaired release of iron from stores or impaired systems for transporting iron to target tissues.

The prevalence of iron-deficiency anemia (10%) represented about two-thirds of the overall anemia prevalence. Also, approximately one tenth (9%) of the children exhibited low VA status, with 0.7% being deficient, and >90% had low serum zinc concentrations, indicating a very high prevalence of zinc deficiency in this population. Hence, this population of school children exhibited concurrent deficiencies of micronutrients, as reported previously for younger children in SE Asia [37], [38].

Poor iron status was associated with lower cognitive ability, however the effect was modified by gender: children with IDA, especially boys, scored lower than iron-replete children. Interestingly, ID was associated with lower scores in the picture completion test even without anemia. Also, lower scores in two of the three tests were found for children with positive but marginal total body iron stores (0–4 mg/kg) as compared to children with replete stores. Thus, the findings from this study indicate that marginal iron status may have impaired cognitive performance before the onset of anemia. This evidence suggests that interventions are needed to improve iron status in school children even in the absence of IDA.

Stunting is an indicator of early-life chronic malnutrition, with most of the growth deficit occurring in the first 2 y of life [39]. In the present study, stunting was a high risk factor for lower scores in all tests after controlling for age, gender and micronutrient status (iron, vitamin A, zinc, iodine), and also for socio-economic status in the sub-sample of children. Furthermore, this study showed that the prevalence of stunting, both moderate and severe, was higher in children ≥10 y than in children <10 y. One possible explanation could be that nutritional status of young children has improved considerably over the last decade in this area, but the national data reported a consistent 40% of stunting among children <5 y in 2005 and 2010 [2], [40]. Another explanation could be that growth faltering continues after 5 y of age. Thus, considering also the extremely high prevalence of zinc deficiency found in the studied 6–16 y children (93%), interventions like zinc fortification could possibly help to limit worsening of stunting and, as a consequence, of cognitive performance impairment.

Children infected by parasites, especially girls, exhibited poorer cognitive performance than non infested children. Possible links between parasite infection and cognitive outcomes are reduced school attendance due to illness, loss of concentration, or through nutrition by affecting absorption of micronutrients such as iron and iodine [41], [42]. In the present study, parasite infections remained a highly significant factor in the multivariate analysis which included anthropometry and micronutrient status indicators, suggesting an effect of parasite infection on cognition independent from nutrition. Therefore, the negative effect of parasite infection on cognition might be associated to higher absenteeism from school due to illness. Regardless of the underlying mechanisms, deworming programs could benefit school performance in Cambodian school children.

Although not available for all studied children, socio-economic status was included in analysis for a sub-sample of children (n = 616). The results obtained from this sub-group were similar to the whole sample and remained unchanged after correcting for confounding effect of caretaker’s level of education and household’ s income. Thus, we can reasonably assume that associations between cognitive outcomes and variables reflecting nutritional status found for the 2443 children are not due to confounding effects of socio-economic status.

Thus, the present study showed that poor cognitive performance was multi-factorial, with nutritional factors as stunting and iron deficiency and non-nutritional factors such as parasite infection being implicated. These results are consistent with recent studies showing a relationship between cognitive abilities below average and low HAZ in school children in South East Asia [13], and a beneficial effect or iron supplementation on attention, concentration and different measures of cognition [5], [6]. Hence, as stunting and iron deficiency in school-aged children reflect long-term and current nutritional status respectively, interventions at different periods of age could have a beneficial effect on cognitive abilities. Nutritional programs in early life to ensure good nutrition and prevent stunting before start of schooling are crucial. In addition, strategies at school-age like deworming, surveys for early recognition and prevention of iron deficiency before the onset of anemia, or interventions to prevent worsening of growth faltering should be considered to contribute to optimal cognitive development. Combining nutrition specific interventions, such as food fortification, with nutrition sensitive interventions, such as deworming, might have synergistic benefits.
